# Targeting mesothelin in ovarian cancer

**DOI:** 10.18632/oncotarget.26350

**Published:** 2018-11-16

**Authors:** Azam Ghafoor, Anish Thomas, Raffit Hassan

**Affiliations:** Thoracic and GI Malignancies Branch, National Cancer Institute, Bethesda, MD, USA

**Keywords:** anetumab ravtansine, ovarian cancer, mesothelin, antibody-drug conjugate, chemotherapy

Mesothelin, a tumor-differentiation antigen present on mesothelial cells of the pleura, peritoneum, and pericardium was discovered at the National Cancer Institute over 20 years ago [1]. It is an attractive target for cancer therapy because of its cell surface location and differential expression between normal tissue and cancer. Mesothelin is highly expressed in many cancers including the majority of ovarian cancers - approximately 70%, and several other tumors including mesothelioma, advanced lung adenocarcinoma, and pancreatic cancer [2-3]. Several mesothelin-directed therapies are now being tested in clinic including anti-mesothelin immunotoxins and antibody-drug conjugates (ADC) [4]. Previous studies by our group and others have shown high mesothelin expression in serous ovarian cancers as well as sensitivity of patient derived cell cultures to mesothelin targeted agents validating it as an attractive tumor type for mesothelin directed therapies [5].

In the September 2018 edition of Oncotarget, Quanz et al [6]. demonstrate the activity of anetumab ravtansine in combination with standard chemotherapies in ovarian cancer models. Anetumab ravtansine is an ADC that contains a human anti-mesothelin antibody conjugated to the maytansinoid tubulin inhibitor DM4 via a reducible disulfide linker [7]. Both *in-vitro* and *in-vivo* studies have demonstrated selective activity of anetumab ravtansine in mesothelin expressing cells and tumors including ovarian cancer [7]. In clinical testing, a phase I trial has determined the safety and maximal tolerated dose of anetumab ravtansine and it is being investigated alone or in combination therapies in several phase I/II trials for patients with mesothelin positive cancers (Table 1) [8].

**Table 1:** Clinical trials of Anetumab ravtansine

Regimen	Cancer type	Combination agent	Year	Phase IIII	ClinicalTrial.gov Identifier:
Anetumab ravtansine	NSCLC	Atezolizumab	2018	IIII	NCT03455556
Anetumab ravtansine	Pancreatic	N/A	2017	II	NCT03023722
Anetumab ravtansine or paclitaxel	Ovarian, Fallopian, Primary Peritoneal	Bevacizumab	2018	II	NCT03587311
Anetumab ravtansine	Solid Tumors	Cisplatin (Cholangiocarcinoma) Gemcitabine (pancreas) N/A (other solid tumors)	2017	Ib	NCT03102320
Anetumab ravtansine	Pleural Mesothelioma	Vinorelbine	2015	II	NCT02610140
Anetumab ravtansine	Pleural Mesothelioma	Pembrolizumab	2018	IIII	NCT03126630
Anetumab ravtansine	Solid Tumors	Pemetrexed and Cisplatin	2016	I	NCT02639091
Anetumab ravtansine	Ovarian, Fallopian, Primary Peritoneal	Pegylated Liposomal Doxorubicin	2016	I	NCT02751918
Anetumab ravtansine	Neoplasms	N/A	2016		NCT02696642
Anetumab ravtansine	Solid tumors	itraconazole	2016		NCT02824042

N/A = Non-applicable

The Quanz et al. studies [6] confirmed the efficacy of monotherapy with anetumab ravtansine both *in vitro* and *in vivo*. Importantly, there was a correlation between the *in vivo* antitumor activity of anetumab ravtansine and mesothelin expression. In combination studies, treatment with anetumab ravtansine resulted in improved anti-tumor efficacy in ovarian cancer models in combination with pegylated liposomal doxorubicin which is approved in the second line for recurrent and platinum resistant ovarian cancers, than with either drug alone. Anetumab ravtansine also showed additive activity with carboplatin – another standard chemotherapeutic agent in first line treatment of ovarian cancer. Furthermore, anetumab ravtansine and the anti-angiogenic agent bevacizumab were determined to be more beneficial in combination than as monotherapy. These pre-clinical data support ongoing and future combination trials of anetumab ravtansine in patients with ovarian cancer such as the ongoing studies with pegylated liposomal doxorubicin and bevacizumab. It will be important for the clinical trials to be adequately powered for efficacy analysis as well as address several important questions such as correlation of anti-tumor activity with mesothelin expression, platinum resistance status, and determining biomarkers of response such as serum mesothelin. Although it has been almost 20 years since the first patient, a patient with ovarian cancer, was treated with a mesothelin targeted agent, these therapies are now starting to show promise in patients and, hopefully, this efficacy will be validated in randomized phase III trials [9].

## References

[1] Chang K (1996). Proc Natl Acad Sci U S A.

[2] Ordóñez NG (2003). Am J Surg Pathol.

[3] Thomas A (2015). Oncotarget.

[4] Hassan R (2016). J Clin Oncol.

[5] Hassan R (2002). Clin Cancer Res.

[6] Quanz M (2018). Oncotarget.

[7] Golfier S (2014). Mol Cancer Ther.

[8] Hassan R

[9] Pastan I (2014). Cancer Res.

